# Checkpoint inhibitor immunotherapy during pregnancy for relapsed–refractory Hodgkin lymphoma

**DOI:** 10.1002/ajh.26527

**Published:** 2022-03-21

**Authors:** Andrew M. Evens, Justin S. Brandt, Cody J. Peer, Tyler Yin, Dale Schaar, Faheem Farooq, Brett Mozarsky, William D. Figg, Elad Sharon

**Affiliations:** ^1^ Division of Blood Disorders Rutgers Cancer Institute of New Jersey New Brunswick New Jersey USA; ^2^ Departments of Medicine and Maternal Fetal Medicine Robert Wood Johnson University Hospital New Brunswick New Jersey USA; ^3^ Clinical Pharmacology Program National Cancer Institute Bethesda Maryland USA; ^4^ Cancer Therapy Evaluation Program, Division of Cancer Treatment and Diagnosis National Cancer Institute Bethesda Maryland USA

## 1 CASE PRESENTATION

**A 31‐year‐old Gravida 2 Para 1 Hispanic female with a history of classic Hodgkin lymphoma (cHL) presented to an outside hospital with fatigue, normocytic–normochromic anemia (hemoglobin 6.0 g/dL), and severe abdominal and lower back pain at 11 weeks gestation. Ultrasound showed large mesenteric and retroperitoneal masses measuring up to 10 cm in diameter. Biopsy at 13 weeks' gestation documented nodular sclerosis cHL.**

**The patient had received 6 cycles of doxorubicin, bleomycin, vinblastine, and dacarbazine (known as ABVD) 3 years prior for initial treatment of stage IVA cHL. She entered clinical remission and had been stable prior to her presentation during pregnancy. Her obstetrical history was notable for a prior full term vaginal delivery, which was complicated by preeclampsia. Her daughter was diagnosed with Klippel–Feil syndrome, a rare skeletal disorder primarily characterized by abnormal fusion of two or more cervical vertebrae.**

**At 19 weeks gestation of the current pregnancy, the patient presented to our center with continued severe fatigue and abdominal pain. The patient's Eastern Cooperative Oncology Group Performance Status was 3, and she had severe anemia with hypercalcemia and other laboratory abnormalities (see Table** **1** **). The patient was admitted to the hospital for urgent medical stabilization. Her multidisciplinary team included hematologic oncology and maternal‐fetal medicine (MFM).**

**TABLE 1:** Laboratory testing (abnormal levels bolded)

Treatment	ESHAP	ESHAP	Nivo	Nivo	Nivo	Nivo	Nivo	Nivo	Nivo/BV	Nivo/BV	BEAM^a^	None	Steroids	Steroids	Steroids	Steroids	None
Gestation (week)	19	22	26	28	30	33	35	37									
Postnatal (week)									3	6	15	17	18	19	20	22	26
CBC																	
Total WBC (3.4–10.8 × 10^3^/μL)	**22.6**	8.0	4.8	**22.9**	**18.7**	**7.1**	7.0	5.4	5.4	4.8	**2.9**	6.1	3.4	10.5	6.7	6.6	4.2
HGB (11.1–15.9 g/dL)	**6.4**	**9.1**	**10.6**	**10.4**	**10.0**	**10.8**	**11.2**	**11.2**	13.0	12.7	**11.3**	**9.1**	**8.6**	**9.5**	**10.0**	**10.1**	**9.9**
Platelets (150–450 × 10^3^/μL)	**675**	395	120	276	237	185	223	191	236	233	198	286	**76**	159	**145**	**120**	**119**
Chemistry																	
Sodium (136–145 mmol/L)	**134**	135	**127**	**134**	**133**	**134**	**135**	**135**	139	139	139	138	**134**	**131**	**134**	**134**	136
Potassium (3.5–5.0 mmol/L)	3.7	3.6	4.0	3.7	4.3	4.1	3.6	4.0	4.0	3.9	3.9	4.0	4.2	4.6	4.4	3.9	3.5
Chloride (98–108 mmol/L)	101	103	100	98	102	105	105	104	104	106	104	102	101	**96**	98	**96**	102
CO_2_ (22.0–33.0 mmol/L)	**20.1**	22.7	**19.0**	22.2	**18.4**	**18.2**	**17.0**	**24.2**	24.2	21.0	24.0	23.8	**20.9**	**20.5**	23.0	**21.2**	24.0
Glucose (70–100 mg/dL)	97	96	**164**	**110**	97	91	**123**	**110**	**110**	**118**	**112**	99	**135**	**327**	**247**	**349**	**121**
BUN (6–23 mg/dL)	20	16	18	12	12	12	15	19	19	19	18	8	16	**28**	**27**	**30**	16
Creatinine (0.5–1.2 mg/dL)	0.7	0.4	0.6	0.5	0.5	0.5	0.6	0.7	0.7	0.5	0.5	0.4	0.4	0.5	0.6	0.5	0.5
Albumin (3.5–5.5 g/dL)	**2.8**	**2.8**	**3.2**	3.6	4.1	3.6	3.7	4.1	4.1	4.4	3.8	3.5	3.7	4.1	4.3	4.1	3.9
Bilirubin total (0.1–1.2 mg/dL)	0.8	0.6	0.9	0.5	0.3	0.2	0.2	0.2	0.2	0.2	0.2	0.3	1.6	1.2	0.7	0.6	0.5
ALT (10–50 IU/L)	40	30	20	30	24	11	7	43	43	36	49	39	**1311**	**787**	**405**	**169**	50
AST (10–55 IU/L)	49	23	27	36	30	18	17	35	35	31	31	31	**463**	**209**	**95**	45	49
Alkaline phosphatase (45–115 IU/L)	**391**	**200**	**407**	**471**	**283**	**114**	**120**	85	85	61	45	**174**	**501**	**524**	**377**	**186**	**281**
Calcium (8.6–10.4 mg/dL)	**12.9**	8.7	**11.0**	9.6	9.4	8.7	8.5	9.0	9.0	9.2	7.8	8.0	9.0	9.4	9.1	9.2	9.

Abbreviations: AL, alanine aminotransferase; AST, aspartate aminotransferase; BEAM, carmustine, etoposide, cytarabine, and melphalan; BV, brentuximab vedotin; CBC, complete blood count; CO_2_, carbon dioxide; ESHAP, etoposide, methylprednisolone (solumedrol), high‐dose cytarabine (ara‐C), and cisplatin; HGB, hemoglobin; Nivo, nivolumab; WBC, white blood count.

^a^ BEAM conditioning chemotherapy given the preceding 5 days.

**FIGURE 1:**
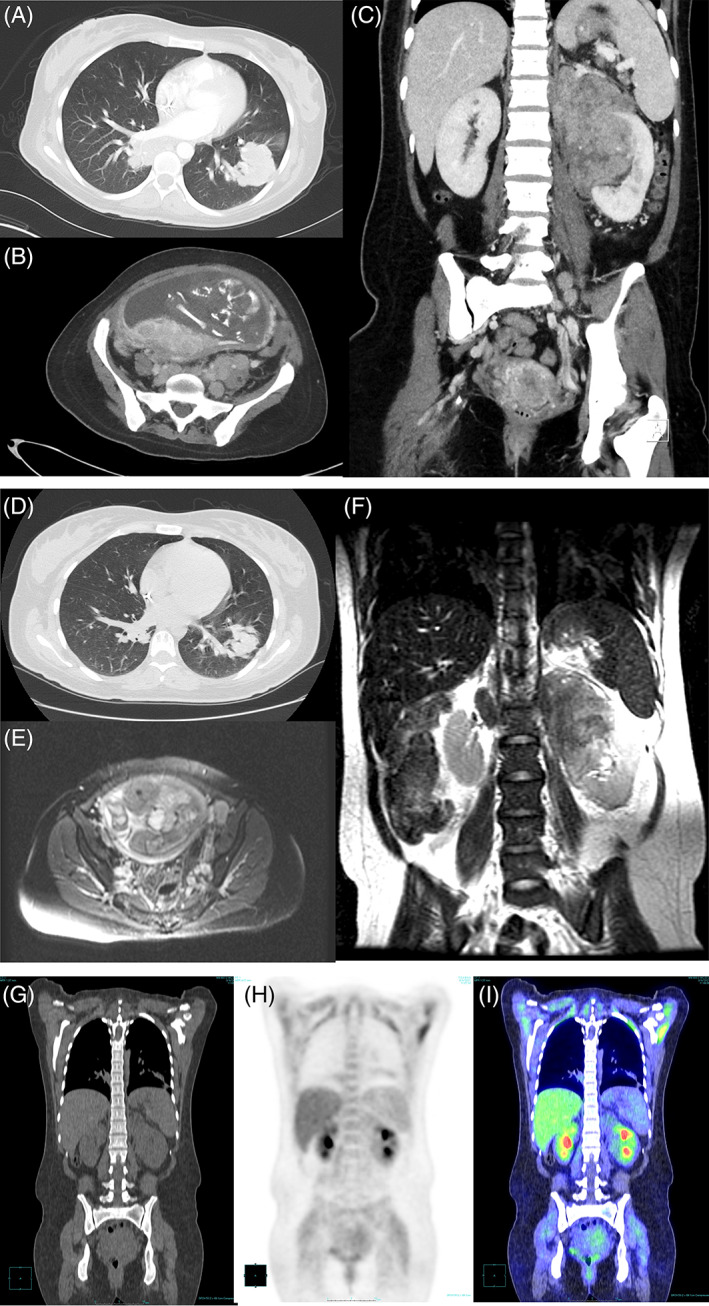
Imaging. (A) CT at 25 weeks gestation of right lower lobe mass measuring 4.5 × 2.4 cm and left lower lobe mass measuring 4.3 × 3.2 cm; (B) CT at 25 weeks gestation with axial view of extensive retroperitoneal and pelvic lymphadenopathy, and gravid uterus; (C) CT at 25 weeks gestation of conglomerate of para‐aortic lymphadenopathy in the upper abdomen with necrotic features measuring approximately 11 × 4.4 cm; (D) CT chest at 32 weeks gestation of right infrahilar location smaller measuring 2.3 × 3.2 cm, and left lower lobe parenchymal mass measured 3.6 × 3.1 cm; (E) MRI abdomen at 32 weeks gestation of axial view of smaller retroperitoneal mass; (F) MRI abdomen at 32 weeks gestation of left sided para‐aortic lymphadenopathy smaller measuring 8.3 × 6.0 × 5.3 cm; (G) PETCT coronal view at 2 weeks postnatal of right lower lobe perihilar opacity measuring 2.2 cm; redemonstration of left renal hilar mass; (H) Coronal re‐formatted PET/CT image at 2 weeks postnatal; and (I) PET/CT at 2 weeks postnatal of fused coronal view with decrease in size of left renal hilar mass at 4.1 cm with low‐level SUV max 1.5. CT, Computerized tomography; MRI, magnetic resonance imaging; PET, positron emission tomography [Color figure can be viewed at wileyonlinelibrary.com]

**The patient received a regimen of modified etoposide, methylprednisolone (solumedrol), high‐dose cytarabine, and cisplatin (ESHAP) chemotherapy with a 50% reduction of cytarabine. She had brisk improvement in her symptoms and laboratory abnormalities (Table** **1** **). A second cycle of modified ESHAP regimen was administered at 22 weeks gestation. However, she developed increased abdominal pain, progressive hypercalcemia, and progressive adenopathy at 25 weeks gestation (Figure** **1** **A–C).**

The management of cancer during pregnancy presents unique medical and ethical challenges that involve balancing maternal and fetal risks associated with either delaying intervention or initiating antenatal therapy. Cancer as a complication during pregnancy occurs in approximately 1 in 1000 gestations, which translates into approximately 4000 cases annually in the United States.^1^ Breast cancers, lymphomas, and dermatological malignancies are among the most common malignancies during pregnancy.^2^

There is increasing evidence that most nonmetabolite chemotherapy can be safely administered during pregnancy, especially beyond the first trimester (i.e., 13 weeks gestation).^3^, ^4^ However, there is a paucity of data regarding the safety and feasibility of treatment with targeted therapeutics and newer immunotherapy agents. With the increasing use of immune checkpoint inhibitors in standard therapy regimens for numerous cancer subtypes, the absence of data for the use of these agents in pregnancy represents a hole in the therapeutic armamentarium for an important and uniquely vulnerable population of patients.

**The patient‐initiated single‐agent intravenous nivolumab at 240 mg at 26 weeks' gestation. There was rapid improvement in the patients' symptoms and laboratory data after one dose of nivolumab. She continued nivolumab every 2 weeks for 6 total antenatal treatments over 78 days. Most doses were given every 14 days, while 21 days elapsed between the third and fourth doses. The trough concentration of nivolumab in maternal blood following the fourth dose was 55.9 μg/mL. Moreover, the mean maternal blood concentration of nivolumab 5 days after the sixth dose was determined to be 84.4 μg/mL, while the mean umbilical cord blood concentration sampled 1 day later was 53.9 μg/mL. No quantifiable nivolumab was measured in the placental tissue.**

**She tolerated checkpoint inhibitor therapy well, and it resulted in a partial remission by computed tomogram and magnetic resonance imaging after 3 nivolumab doses at gestation 32 weeks (Figure** **1** **D–F). She received her final antenatal nivolumab dose at 37 weeks gestation. The patient had induction of labor at 38.2 weeks and had a vaginal delivery of a male neonate. Her delivery was uncomplicated with a first‐degree perineal laceration and estimated blood loss of 300 cm^3^. The birth weight was 2315 g; birth length was 17 cm. Postnatal evaluation of the neonate was normal. The neonate had no laboratory abnormalities during a routine hospitalization of 2 days.**

**A restaging positron emission tomography–computed tomogram 2 weeks postnatal confirmed complete metabolic remission (Figure** **1** **G–I). Based in part on patient wishes, she had a 2‐month delay before proceeding with autologous stem cell transplant (ASCT). The patient received two cycles of nivolumab and brentuximab vedotin therapy intravenously at 3‐ and 6‐weeks postnatal and additional single‐agent brentuximab vedotin, which she tolerated well. She had carmustine, etoposide, cytarabine, and melphalan conditioning chemotherapy with the ASCT at 15 weeks postnatal, and was discharged day + 13 ASCT at 17 weeks postnatal with near full count recovery.**

**However, the patient was readmitted to the hospital 1 week later with severe malaise, nausea, and highly elevated liver function tests (Table** **1** **). Workup was overall negative, including abdominal ultrasound with dopplers, and detailed infectious workup. Flow cytometry of the peripheral blood showed an expanded NK cell population (41%); 17% of these lymphocytes were CD3 positive T‐cells without significant loss of pan‐T‐cell antigens or downregulation and CD4:CD8 ratio of 0.4:1. The remaining cells were surface CD3 negative, CD56 positive, NK cells without definitive aberrant immunophenotype.**

After the discovery of relapsed cHL during pregnancy in this patient, the patient faced a failure of a well‐established, standard chemotherapeutic regimen with a continued progressive disease that was life‐threatening for the patient and the fetus. After counseling about potential benefits and risks,^5^ including lack of safety data in pregnancy, the patient proceeded with single‐agent checkpoint inhibitor therapy given its well‐established track record in cHL.^6^

Due to physiologic adaptations of pregnancy, the optimal dose of chemotherapy or other anticancer treatments during pregnancy remain unclear. Fetal uptake of anticancer agents can also be altered by the changes in uterine and umbilical blood flow, placental thickness, and placental transfer. Another important concern is lower plasma drug exposure due to increased plasma volume, glomerular filtration rate and renal clearance, and hepatic oxidation.^7^, ^8^ These physiologic adaptations may affect plasma and tumor concentrations of chemotherapy, thereby reducing chemotherapeutic efficacy.^9^, ^10^

**FIGURE 2:**
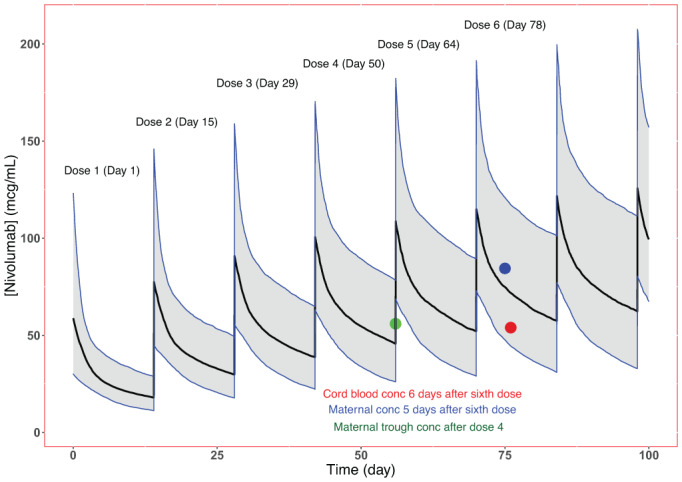
Observed and simulated nivolumab serum concentrations. The observed concentrations measured in serum obtained from maternal peripheral blood (predose, green; postdose, blue) and the umbilical cord blood (red dot) were overlaid on simulated serum concentration versus time following a 240 mg every 2‐week regimen (median [90% prediction interval], black line [shaded region]). The patient underwent spontaneous labor 6 days following the sixth nivolumab dose. The observed data from the mother were very close to the median expected values. The umbilical cord concentrations indicated nivolumab was able to cross the blood‐placental barrier. No nivolumab was detected in the placental tissue [Color figure can be viewed at wileyonlinelibrary.com]

To understand if the maternal serum concentrations of checkpoint inhibitor therapy were within expected ranges, population pharmacokinetic (PK) simulations from 1000 virtual patients administered with nivolumab via intravenous dosing at 240 mg every 2 weeks were performed. The cycle 4 trough value (55.9 μg/mL) fell within the 80% prediction intervals generated by the 240 mg q 2 weeks nivolumab PK simulations (Figure 2). Both observed concentrations in maternal and umbilical cord blood following the sixth dose were consistent with the simulated nivolumab serum concentrations as well.

It should also be noted that despite a 9‐week interval between the patients' final checkpoint inhibitor dose and the ASCT that this therapy may have played a role in her engraftment syndrome, in addition to the patients' postpartum immune milieu.

**The patient was started on high‐dose pulse steroids for presumed engraftment syndrome post‐ASCT. She had steady improvement with ultimate resolution of her liver function abnormalities, and steroid therapy was concomitantly weaned off over a 6‐week period (Table** **1** **). The patient has remained in complete remission 9 months post‐ASCT (** **Appendix** **S1).**

## 2 DISCUSSION

The decision to initiate anticancer therapy during pregnancy is highly personalized involving the collaboration and guidance of a multidisciplinary team with each patient weighing potential adverse effects of fetal exposure during treatment against the potential benefits for the patient and fetus. The multidisciplinary team should include hematology–oncology, MFM, neonatology, and other relevant subspecialties. Factors that need to be considered in the selected treatment plan include gestational age, subtype of lymphoma, symptomology, and anatomic sites of disease.^11^

It is important to highlight that a term delivery (i.e., gestational age >37 weeks) is preferable to iatrogenic preterm delivery for most cases as gestational age drives neonatal outcomes and prematurity is associated with increased risk for complications, such as respiratory distress syndrome, necrotizing enterocolitis, and intracranial hemorrhage of the newborn. In a cohort of 70 treated women during pregnancy with 236 cycles of chemotherapy, cognitive development scores were lower for children who were born preterm with an 11.6 point lower intelligence quotient for each additional month of preterm delivery.^12^

There have been reports in the medical literature of the use of immune checkpoint inhibitors for patients with cancer with unexpected pregnancy,^13^, ^14^, ^15^, ^16^, ^17^, ^18^ including patients who were on clinical trials when pregnancy was discovered.^19^ However, this is the first known case of a lymphoma patient treated with an immune checkpoint inhibitor during pregnancy and the only report of this therapy given past week 33 of gestation. While the patient's postnatal outcome included severe engraftment syndrome, this resolved with supportive measures and she has no evidence of disease at present. The resulting offspring appears similarly unaffected by antenatal therapies administered.

Given the marked activity of PD‐1 inhibitors in cHL and the use of these agents in other malignancies, it is important to gather data on patients who may become pregnant or discover they are pregnant when the use of these agents would be otherwise warranted. Well‐intentioned policies by ethics boards in medicine have consistently referred to pregnant patients as a vulnerable population. This leaves patients facing cancer with two options: terminate an unexpected pregnancy or continue therapy with potentially less effective treatments developed decades ago, often prior to the advent of these policies.

Maternal immune tolerance is maintained in part via the PD1/PD‐L1 pathway, with PD‐L1 expression particularly enriched in gestational trophoblasts and regulatory T‐cells at the fetal–maternal interface. Anti‐PD1/PD‐L1 administration has been shown to increase the risk of spontaneous abortion in rodent models and is categorized as category D.^20^ However, there is insufficient evidence of an increased risk of either spontaneous abortion or perinatal toxicity associated with exposure to this class of agents in humans.^21^ As this case confirms, there is likely fetal exposure when a monoclonal antibody of an IgG subtype is used therapeutically in a pregnant patient, in line with the known level of maternal‐fetal passive antibody transfer. It should be noted that placenta permeability varies with the IgG subtypes^22^ and cannot be extrapolated to other checkpoint inhibitors. In addition, the fetotoxic effect of anti‐PD1/PDL1 might be patient‐specific varying with the antigenicity of the fetus.

From an evolutionary perspective, such antibody transfer likely protects the developing fetus from infectious pathogens for which an immature fetal immune response would be ineffective. It is, therefore, reasonable to be concerned about the potential for therapeutic antibodies to cause fetal harm. However, this passive antibody transfer would likely need a target in the recipient fetal host to cause said harm. In the case of PD‐1 inhibitors, it appears that the immature fetal immune system itself proves to be protective, with regard to agents that modulate T cell immunity in order to mediate therapeutic effects.

Altogether, while case reports have been published exhibiting treatment with immune checkpoint inhibitors during pregnancy, these were often unintentionally exposed individuals, and none were treated past 33 weeks' gestation. T cell‐modulating therapies may offer an effective therapeutic option for patients with cancer during pregnancy, while maintaining some margin of safety for the developing fetus, despite likely maternal‐fetal transfer of PD‐1 inhibiting monoclonal antibodies, which was confirmed in the cord blood of the fetus in this case. Regardless, signfiicantly more clinical and translational research is needed in this important area of unmet need to bring modern precision oncology tools and targeted therapies to pregnant patients with cancer.

## CONFLICT OF INTEREST

Andrew M. Evens: Advisory Board (research related): Seattle Genetics, MorphoSys, Karyopharm, Novartis, Pharmacyclics, Abbvie, and Epizyme; all other authors: none.

## Supporting information


**Appendix S1**. Supporting information.

## Data Availability

The data that support the findings of this study are available from the corresponding author upon reasonable request.
