# Supplementary material for: Checkpoint inhibitor immunotherapy during pregnancy for relapsed–refractory Hodgkin lymphoma
Source: Am J Hematol. 2022 Mar 21;97(6):833–8. doi: 10.1002/ajh.26527 (PMC9314600; doi:10.1002/ajh.26527)
Supplement: Supplementary file 1 — Appendix S1. Supporting information. [file AJH-97-833-s001.docx]

**Methods**

*Nivolumab Dosing Regimen*

Treatment of relapsed Hodgkin’s Lymphoma with nivolumab was initiated at 26 weeks gestation using a 240 mg intravenous dosing regimen every two weeks. The pregnant female patient received six doses prior to delivery: December 1^st^, 2020 (day 1, dose 1), December 16^th^, 2020 (day 15, dose 2), December 30^th^, 2020 (day 29, dose 3), January 20^th^, 2021 (day 50, dose 4), February 3^rd^, 2021 (day 64, dose 5), February 17^th^, 2021 (day 78, dose 6).

*Patient Blood Collection and Placental Tissue Extraction*

Patient blood samples were collected at specific timepoints over the course of the nivolumab dosing regimen. After initiation of therapy, a blood and tissue collection protocol was written and sent to the Rutgers University Institutional Review Board for patients who experience cancer during gestation. The patient was apprised of the minimal risks associated with participating on the trial and consented to contribute maternal blood, placental tissue, and umbilical cord blood to the trial.

Maternal blood was sampled just prior to the fifth dose of nivolumab (February 3^rd^, 2021; day 64) and five days after the sixth dose (February 22^nd^, 2021; day 83). Umbilical cord blood and placental tissue were both obtained on the day of delivery (February 23^rd^, 2021; day 84), six days after the sixth dose. For each timepoint, whole blood was collected into serum separator tubes (SST) and allowed to clot for 30 minutes at ambient (room) temperature. Serum (1 mL) was drawn from the clotted blood, added to a 9 mL PBSTF solution (0.1% Tween®-20 and 1% Ficoll-Pacque™ PLUS in PBS), and mixed vigorously. The 10 mL solution of 10% serum/PBSTF was further aliquoted into ten, 1-mL Nunc cryovials and frozen at -80 °C. Placental tissue was removed from the maternal surface, the fetal surface, and the middle parenchyma and snap frozen.

*Preparation of Patient and Umbilical Cord Blood and Placental Samples*

Study samples from maternal or cord blood were shipped and stored in 10% serum/PBSTF. At the time of bioanalysis, samples were thawed and diluted 10-fold to match the standards (1% serum/PBSTF), then analyzed via ELISA. Placental samples were weighed and homogenized in water to a homogenate concentration of 100 mg/mL. This homogenate was diluted 100-fold to a 1% homogenate in PBSTF, then analyzed via ELISA.

*Enzyme Linked Immunosorbent Assay (ELISA)*

All aliquoted patient samples were quantitated using a CLIA-level certified and validated ELISA assay with a range of 25-800 ng per mL. Standards and QCs were aliquoted in duplicate and triplicate, respectively, across the microplate coated with PD-1 (purchased from BioVision, Milpitas, CA). Blanks containing only 1% serum/PBSTF vehicle matrix were aliquoted in duplicate, while patient samples were aliquoted in triplicate. Drug-free, control human placental tissue (BioIVT, Baltimore, MD) was spiked with the appropriate concentration of nivolumab for a given standard or QC, homogenized at 100 mg per mL in 18.2 MΩ∙cm water, and diluted to 1% homogenate/PBSTF to allow for comparisons with other samples in a 1% serum/PBSTF vehicle matrix.

Standard curves were constructed by plotting the log-transformed standard concentrations versus the average OD_450nm_ for standard calibrators using a nonlinear regression of log (agonist) versus response function model. The logarithms of measured nivolumab concentrations were calculated and the anti-log of each value was taken to determine drug concentration. After multiplying each concentration by an appropriate factor to correct for sample dilution, the observed nivolumab concentrations for standards and QCs were compared to the corresponding expected concentrations. In all passing ELISA assays, the observed concentration of each calibration standard and QC was within 20% of its expected concentration (25% allowed).

*Population PK Simulations*

A population PK by Liu et al was replicated and used for simulations of 240 mg every two weeks.^6^ One thousand unique values for each of the included covariates in the model were randomly generated using R v4.1 (www.r-project.org). The predicted nivolumab serum concentrations were plotted using ggplot2 package in R, with the median (50^th^ percentile) and the 80% prediction interval presented. Observed data from this study were overlaid with simulations.
